# Microbubble-Enhanced Focused Ultrasound for Infiltrating Gliomas

**DOI:** 10.3390/biomedicines12061230

**Published:** 2024-06-01

**Authors:** Alexandra A. Seas, Adarsha P. Malla, Nima Sharifai, Jeffrey A. Winkles, Graeme F. Woodworth, Pavlos Anastasiadis

**Affiliations:** 1 Department of Neurosurgery, University of Maryland School of Medicine, Baltimore, MD 21201, USA; 2 Marlene and Stewart Greenebaum Comprehensive Cancer Center, University of Maryland Medical Center, Baltimore, MD 21201, USA; 3 Department of Pathology, University of Maryland School of Medicine, Baltimore, MD 21201, USA

**Keywords:** high-grade gliomas, glioblastoma, infiltrating gliomas, focused ultrasound, blood-brain barrier, immunotherapy, microbubbles, acoustic emissions

## Abstract

Infiltrating gliomas are challenging to treat, as the blood-brain barrier significantly impedes the success of therapeutic interventions. While some clinical trials for high-grade gliomas have shown promise, patient outcomes remain poor. Microbubble-enhanced focused ultrasound (MB-FUS) is a rapidly evolving technology with demonstrated safety and efficacy in opening the blood-brain barrier across various disease models, including infiltrating gliomas. Initially recognized for its role in augmenting drug delivery, the potential of MB-FUS to augment liquid biopsy and immunotherapy is gaining research momentum. In this review, we will highlight recent advancements in preclinical and clinical studies that utilize focused ultrasound to treat gliomas and discuss the potential future uses of image-guided precision therapy using focused ultrasound.

## 1. Introduction

Gliomas are primary brain tumors derived from neuroglial precursor cells and are commonly stratified by histological and molecular features into astrocytomas, oligodendrogliomas, ependymomas, and other/rare types [1]. They are further assigned WHO grades 1–4 based on clinical phenotype and prognosis [2]. Gliomas of WHO grades 3 and 4 are classified as high-grade gliomas (HGGs) and include glioblastoma (GBM) and pediatric gliomas such as diffuse intrinsic pontine glioma (DIPG) [3]. GBM is the most common primary adult malignant glioma, accounting for >50% of all gliomas, and is the most aggressive and heterogeneous glioma—it is uniformly fatal, with a median survival of less than 18 months [1,4,5].

Gliomas are typically diagnosed by tissue biopsy after the onset of neurological symptoms such as seizures or cognitive disorders [6]. Conventional magnetic resonance imaging (MRI) is a critical tool in glioma diagnosis and disease monitoring, as it provides robust structural information. Imaging features such as contrast enhancement, vascularity, and tumor mass effect may be indicators of HGGs; however, these are not specific markers. More advanced imaging techniques such as diffusion-weighted imaging, diffusion tensor imaging, connectomics, spectroscopy, and positron emission tomography have been utilized in clinical practice and clinical trials—these techniques can potentially elucidate functional properties of gliomas [7,8,9]. Despite these advancements, conventional MRI remains the core radiologic diagnostic tool for gliomas.

Standard care for patients with high-grade gliomas is a maximally safe surgical resection with adjuvant radiotherapy targeting the tumor margin and invasive rim. Treatment of GBM often includes adjuvant chemotherapy with the alkylating agent temozolomide (TMZ). While adding TMZ confers some survival benefits for GBM compared to adjuvant radiation alone, it does not result in significant progression-free survival [10,11]. Moreover, GBM is notorious for intrinsic and acquired resistance to chemotherapy and radiation, further limiting the effectiveness of these therapeutic interventions. In recent years, there has been a significant push towards developing novel therapies to enhance survival and methods for improving the drug delivery of currently available therapies [12].

Gliomas, particularly GBM, are challenging to treat for several reasons. First, their infiltrative nature makes complete surgical removal nearly impossible, even with supramarginal resection. Surgical treatment is further complicated by the fact that these tumors frequently invade eloquent brain areas such as the motor and somatosensory cortices, making gross total resection of just the contrast-enhancing components high-risk for neurological deficits [13,14]. Secondly, while immunotherapies have shown promise in treating other malignancies, the unique immune landscape of the brain poses challenges for immunotherapy applications in glioma treatment [15,16]. Finally, the blood-brain barrier (BBB) is a significant impediment to effective glioma treatment. The BBB is a neuro-vascular interface between the systemic blood circulation and the brain parenchyma. The BBB plays a critical role in protecting the central nervous system (CNS) from systemic insults and preserving homeostasis in the brain. However, it also hinders the trafficking of exogenous molecules (e.g., therapeutics) into the brain [17]. BBB function depends on the neurovascular unit comprised of multiple cell types: microvascular endothelial cells, pericytes, and astrocytes (specifically the foot processes). The movement of molecules across the BBB is primarily regulated by tight junctions formed between endothelial cells, which block paracellular transport. Pericytes line this border, forming part of the extracellular matrix. Astrocyte foot processes form a complex network outside the pericytes, which connect the vascular compartment and the neuroglial network. The foot processes significantly maintain ionic and metabolic homeostasis at the BBB [18]. The BBB also contributes to the tight regulation of brain–immune interactions. It shields the brain from circulating toxins and protects fragile brain parenchyma from the potentially deleterious effects of robust systemic immune responses [16,19].

Due to the restrictive BBB and an immunosuppressive tumor microenvironment, a large portion of CNS tumors are shielded from immune surveillance; that is, the host immune system is unable to identify the tumor as foreign failing to mount an effective immune response, even when it is stimulated with pharmaceutic agents [20,21]. Several methods have been developed to bypass the BBB, both to enhance the delivery of therapeutic agents and to allow for immune surveillance of the brain. Focused ultrasound (FUS) is one such method that has garnered significant attention in neuro-oncology over the past two decades.

## 2. Focused Ultrasound (FUS)

FUS is a rapidly evolving technique in which acoustic waves are targeted to a specific area. FUS was first described as a method for therapeutic tissue heating or thermoablation [22]. Tissue heating is thought to be helpful in the context of treatment-resistant tumors and has shown efficacy in neurological and movement disorders such as Parkinson’s disease. FUS has also been explored as a potential treatment for chronic pain and carcinomatosis and has been utilized as an alternative to lobotomy [23].

**Figure 1:**
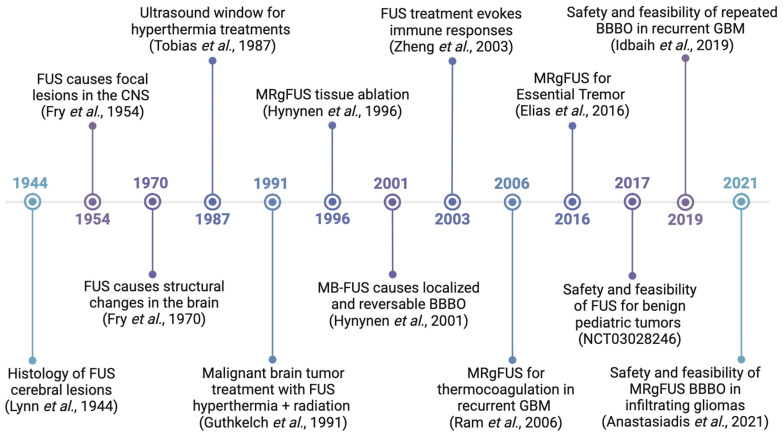
**A brief timeline of FUS BBBO history.** This timeline shows critical events that have led to current research and clinical trials using FUS. Adapted from the Focused Ultrasound Foundation Timeline of Focused Ultrasound. Created with BioRender.com [24,27,30,31,32,33,34,35,36,37,38,39].

The history of FUS for brain pathologies dates back to the 1950s, when the brothers William and Francis Fry developed the first FUS system for creating focal lesions in feline brains. Their novel technique did not affect surrounding tissues or delicate vasculature, mitigating the central issue associated with invasive surgical ablation [24,25]. The Fry brothers also established the reversibility of low-power FUS-mediated neuroanatomical changes [26] and determined threshold ultrasound doses for varying levels of neuromodulation in mammalian brains [27]. These discoveries led the Fry brothers to spearhead clinical studies of FUS to treat brain pathologies [28]. However, their work was limited by the need for a cranial window to limit the phase distortion of the ultrasound waves. The development of phased array systems in the late 1990s enabled the use of phase corrections to overcome aberrations caused by the skull without the need for cranial windows [29], ultimately leading to a substantial increase in neurological research using FUS (Figure 1). A landmark trial applying FUS for thermal ablation in Essential Tremor led to the FDA approval of the first FUS brain device in 2016 [30].

**Figure 2:**
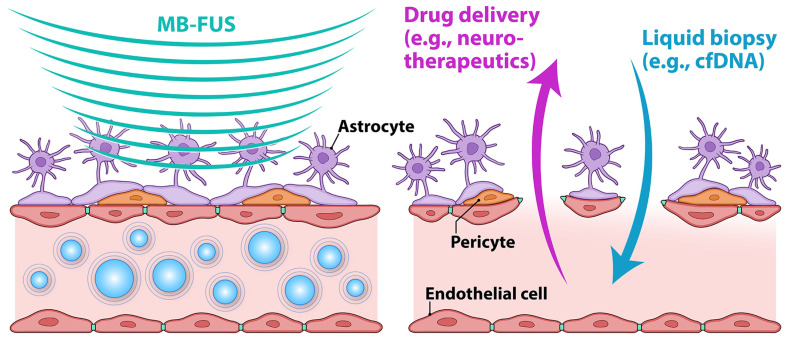
**Effects of MB-FUS on the BBB.** The schematic depicts the BBB with the brain parenchyma above and the blood vessel with circulating MBs below (**left**). Note the cell types of the neurovascular unit: endothelial cells, pericytes, and astrocytic endfeet. MBs oscillate as they enter the acoustic field. At low MB-FUS intensities, typical for MB-FUS BBBO, the MBs oscillate stably, leading to the transient disruption of endothelial tight junctions, which then, in turn, lead to BBBO (**right**). MB-FUS BBBO allows small molecules to pass through the BBB for enhanced drug delivery (purple arrow) or tumor-associated DNA to enter the bloodstream for liquid biopsy (blue arrow).

More recently, attention has been given to the potential of FUS for BBB opening (BBBO) in neuro-oncology, specifically with the addition of microbubbles (MB). MBs are micron-sized gas-filled bubbles originally developed as imaging contrast agents. Upon interaction with an acoustic field, MBs undergo oscillations, which, in turn, cause mechanical perturbation of endothelial-cell tight junctions, resulting in transient BBBO (Figure 2). MBs are injected intravascularly into the bloodstream as a single bolus or a continuous infusion during FUS treatments. The interactions between MBs and FUS are tunable depending on the frequency and power of FUS beams and the dose of MBs. Furthermore, the extent of BBBO can be customized depending on the expected result and safety controls.

Landmark studies of microbubble-enhanced FUS (MB-FUS) for BBBO used MRI guidance to treat only certain brain areas—termed MR-guided FUS (MRgFUS). Successful BBBO was confirmed by new signal intensity in the brain parenchyma on contrast-enhancing MRI [34]. This effect was proportional to the MB dose and FUS intensity—a higher dose was analogous to greater signal intensity. Follow-up MRI scans in preclinical and clinical studies have shown that the BBB returns to its baseline within several hours [34,40,41]. Recent studies have indicated the potential of MB-FUS for radiation sensitization in glioma, further expanding the utility of this technology [42].

One of the most common adverse effects of MB-FUS is the formation of microhemorrhages in the brain. This can be seen on MRI as hypointense (dark) puncta on susceptibility-weighted sequences [43]. Microhemorrhages are seen more commonly with increasing ultrasound intensity and increased MB dose, corresponding to increased degree of BBBO. However, studies have shown that despite several areas of brain microhemorrhage, there are no effects on long-term cognition and neurological function [44,45,46]. Comparative MRI imaging shows that these microhemorrhages often resolve; thus, repeated FUS treatments can be performed without permanently damaging brain tissue [47].

Following initial studies indicating reversible BBBO using FUS and MBs, research shifted towards establishing the safety and efficacy of this process. Histological studies showed the expected vascular effects of FUS without ischemia or apoptosis of neuronal cells up to several weeks after treatment, further indicating that FUS can achieve targeted BBBO without damaging the brain [48]. Furthermore, groups have aimed to establish parameters to induce BBBO without tissue heating by altering ultrasound burst length, pulse repetition frequency, and MB dose [49,50]. Kinetic studies with dynamic contrast-enhanced MRI confirmed increased BBB permeability as a function of the ultrasound strength and MB dose compared to untreated groups [51]. Several other studies laid the groundwork for characterizing MB-FUS BBBO by analyzing the effects of phase distortion, cavitation response, pharmacological delivery, MB size, and MB acoustic emissions on BBBO [31,52,53,54,55,56,57].

## 3. Preclinical Applications of FUS

### 3.1. Drug Delivery

Given the safety and reversibility of BBBO with FUS, it has been investigated as a potential avenue for enhancing GBM drug delivery. Studies have shown that MB-FUS can increase small-molecule-drug transmembrane transport when it is injected just before or immediately after treatment [58]. Drug delivery across the BBB also depends on the size of the agent to be delivered; particularly small and particularly large agents do not cross the BBB as easily as moderately sized ones (tens of nanometers in diameter) [59]. Thus, FUS provides the potential for glioma therapy with small molecules (nanometers in size), nanoparticles, and even chimeric antigen-receptor T cells [60,61].

#### 3.1.1. Drug Loaded MBs

As previously discussed, BBBO with MB-FUS depends on gas-filled MBs, which oscillate upon interacting with the acoustic energy field (Figure 2). MBs are gas–liquid emulsions with a gaseous core that is stabilized by a surrounding shell comprising lipids, proteins, or other biocompatible molecules. The size of MBs allows for the loading of sufficient drugs of interest and the conjugation of targeting moieties for an additional dimension of targeted delivery [62]. Additionally, drugs can be dissolved within the oil layer of the shell or directly incorporated into the shell, which can protect the cargo from degradation and clearance, as well as minimize off-target toxicity. However, a technical concern exists with drug-loaded MBs in that the disruption of the MB structure is required for drug release, which would only be secondary to inertial cavitation induced by the application of higher ultrasound pressures. This could raise concerns about the unwanted effects of MB cavitation, such as microhemorrhages or gliosis. The majority of these studies apply drug-loaded MBs in conjunction with FUS-BBBO by loading these microspheres with chemotherapeutics such as carmustine (BCNU) [63,64], doxorubicin [65,66], or cabazitaxel [67]. Few of these studies also applied an MB-based targeting strategy in conjunction with drug loading. Most studies found that MB-FUS BBBO combined with drug-loaded MBs increased drug accumulation within the targeted tissue and extended animal survival without inducing unstable microbubble cavitation or hemorrhage.

#### 3.1.2. Free Drugs

With the establishment of MB-FUS to increase drug delivery, initial efforts focused on delivering compounds with known pharmacologic properties across the BBB. Seminal studies utilized TMZ, the standard-of-care chemotherapy for GBM and other gliomas. These studies showed that FUS treatment before drug administration improved the accumulation and retention of TMZ in the tumor space, reduced tumor progression, and enhanced survival in animals [68,69,70]. MB-FUS has also opened a door for exploring pharmacological agents that previously were ineffective at treating gliomas. These include platinum agents such as carboplatin, anthracyclines such as doxorubicin, and several others [71,72,73,74,75]. These trials indicated that administration of MB-FUS treatment, as well as administration of the drug of choice, enhanced survival and reduced progression of gliomas, further confirming the challenge of using the BBB to treat CNS tumors. Given promising results in preclinical trials, several of these drugs are currently being explored in clinical trials of FUS for glioma treatment [76,77,78].

#### 3.1.3. Nanomedicines

Nanomaterials have been explored in drug delivery for several years and have been utilized for varying purposes, including improving drug solubility and biodistribution, decreasing degradation, and targeting drugs to areas of interest. These highly customizable systems allow for more robust control of drug release into the tumor environment [79]. Nanostructures were previously underutilized in the treatment of glioma because standard formulations are too large or possess morphological features that limit their ability to bypass the BBB regardless of targeting strategies [80]. The development of MB-FUS BBBO expanded the utility of nanomaterials for glioma treatment. Many trials have focused on novel methods of packaging known and verified anticancer drugs to increase their stability and provide a method of controlled and prolonged drug release—this includes liposomal, polymeric, and peptide encapsulation. Studies using these methods showed increased antitumor efficacy and prolonged survival in animal glioma models [81,82,83]. Other than drug encapsulation, nanostructures can also be used as drug conjugates to increase penetration and targeting to the brain. Promising conjugates in glioma treatment include inorganic compounds such as gold nanoparticles and organic conjugates such as polymers of hyaluronic acid, albumin, and engineered DNA structures [84,85,86,87].

#### 3.1.4. CAR-T Cells

Adoptive immunotherapies, such as CAR-T cells, have revolutionized cancer treatment for certain hematological malignancies and solid tumors. However, the success of CAR-T cells for primary brain tumors has been minimal due to several challenges associated with GBMs, such as the BBB and an immunosuppressive microenvironment. Only one study to date has reported the effects of MB-FUS on CAR-T cell trafficking, persistence, and efficacy [88]. This study directly compared CAR-T cell delivery and persistence with and without MB-FUS BBBO and observed increased delivery with MB-FUS BBBO at 24 and 72 h after administration. Additionally, in tissues treated with MB-FUS BBBO, they observed an enrichment of the CAR-T cells 15 days after treatment. This improved delivery also had biological significance, as CAR-T cells delivered with MB-FUS BBBO extended median survival by >50 days compared to the no-MB-FUS BBBO control group. Still, as has been observed in humans and preclinical studies with CAR-T cells, neurotoxicity, and immune-related adverse effects are serious concerns. Additional preclinical studies will be critical to determine whether MB-FUS can enhance the delivery and efficacy of this immunotherapy approach while reducing off-target effects.

### 3.2. Sono-Liquid Biopsy

FUS has been explored more recently in the context of augmenting liquid biopsy (LBx). This is a method that involves the collection of patient blood and analysis for biomarkers. The use of LBx in oncology has expanded dramatically in the last 10 years for early disease detection, therapy guidance, residual disease, and outcome monitoring [89]. In brain tumors, the utility of LBx has been impeded by the BBB, which prevents tumor DNA and tumor-specific biomarkers from entering the circulation. Recent studies have shown that MB-FUS BBBO treatment can increase plasma levels of brain-specific biomarkers and tumor-associated DNA in mouse and porcine GBM models [90,91,92]. Data from these studies support the clinical translation of MB-FUS BBBO-mediated LBx to diagnosis or disease monitoring [93], and a prospective clinical trial has proven the safety and efficacy of this process, as well as proving that sonobiopsy increased levels of circulating tumor DNA in HGG patients [94].

### 3.3. Immune System Modulation

The effects of FUS on the immune microenvironment of the brain have also opened new avenues for research in the context of immune-system modulation. It was previously understood that brain tumors such as gliomas are generally shielded from the immune system [95]. This limits the use of immunotherapies that have been impactful in other areas of oncology, such as immune checkpoint inhibitors, adoptive T-cell therapy, and therapeutic vaccines [96]. MB-FUS BBBO treatments have been shown to induce mild and transient neuroinflammation in animal models, indicating that the process of opening the BBB may allow for immune surveillance of the brain tissue [97]. This may convert the previously immunosuppressive tumor microenvironment to an immune-activated one [98]. In addition to its transient inflammatory effects, FUS treatment in a mouse glioma model has been shown to upregulate specific markers of innate and adaptive immunity within the tumor microenvironment, even showing dependence on FUS intensity and MB dose [99,100]. When combined with known immunotherapies such as anti-PD1, IL12, and anti-CD47, FUS improved antitumor immune response and, in some cases, extended survival in glioma animal models [88,101,102].

### 3.4. Sonodynamic Therapy

A novel application of FUS is selective acoustic activation of therapeutics (sonodynamic therapy (SDT). SDT utilizes sonosensitizing agents, which are delivered to the tumor microenvironment and which, when activated by FUS waves, have therapeutic effects. This has the potential to limit the off-target toxicities of some glioma treatments and provide a different avenue for noninvasive treatment of gliomas [103,104]. Early studies have focused on using 5-aminolevulinic acid, fluorescein, and TMZ as sonosensitizers in murine and porcine glioma models. These have yielded promising preliminary data for increased accumulation of the sonosensitizer within the tumor microenvironment, as well as evidence of safety—animals were not found to have damage to surrounding brain tissue following SDT [105,106,107,108,109].

## 4. Clinical Applications of FUS

Currently, 24 interventional clinical trials registered with ClinicalTrials.gov utilize MB-enhanced focused or unfocused ultrasound to treat gliomas. Most of these trials use one of four FDA-approved clinical-grade ultrasound systems (Table 1). Each system offers a fully customizable spectrum of treatment for each patient, and each is unique in its application.

### 4.1. Comparison of Clinical FUS Systems

InSightec’s Exablate model 4000 Type II system comprises a phased array helmet-like apparatus with 1024 transducers, which can be tuned with MRI-guidance for transcranial sonication of foci in the brain. While providing anatomical guidance, MRI thermometry is also utilized to track temperature in the focal region for treatment adjustment [110]. In addition to being studied for BBB disruption, the Exablate Type I (650 kHz) system has also been FDA-approved for the treatment of essential tremor and Parkinson’s disease [111,112]. A similar experimental system was first utilized in clinical trials for BBBO in non-human primates and ALS patients, wherein MRgFUS BBB disruption was validated radiographically by gadolinium contrast, and this disruption was resolved mainly within 24 h [44,113]. These studies elucidated the potential role of MRgFUS with the Exablate system for BBB opening and subsequent drug delivery [44]. In the context of glioma, the Exablate Type II (220 kHz) system is being utilized in several clinical trials of GBM and recurrent glioma for safety and feasibility, as well as for the potential increase in drug delivery for these patients following maximal bulk tumor resection.

The NaviFUS system and Cordance Medical NeuroAccess devices utilize multiple elements to facilitate FUS treatment. Both use pre-treatment CT/MRI images with patented protocols to localize the area of interest and upload it to the device. The NaviFUS system uses a multi-channel hemispherical phased array ultrasound connected to a flexible arm and intra-procedure neuronavigation tracking for focal guidance. Furthermore, real-time passive cavitation data are collected to monitor energy levels and patient outcomes [114]. The NeuroAccess system, on the other hand, utilizes the ‘Cordance Cap’, a multi-transducer helmet that is specially fitted to each patient and connected to a monitor. NeuroAccess is also the first system indicated for enhancing liquid biopsy in those with brain pathologies [94,115].

The CarThera Sonocloud-9 device is unique to the other three systems in that it is implanted into a skull window, harkening back to early FUS methods used to bypass the mechanical barrier of the intact skull. It comprises 9 ultrasound transducers and utilizes fixed low-intensity pulsed ultrasound (LIPU). Phase 1 studies have shown that when it was activated in conjunction with circulating MBs, the Sonocloud-9 device was able to facilitate transient BBBO and increase parenchymal delivery of albumin-bound paclitaxel (Abraxane) and carboplatin. Additionally, imaging showed that the BBB opening diminished within one hour following LIPU treatment, highlighting the transient nature of MB and ultrasound-mediated BBB opening [78].

### 4.2. Review of Clinical Trials

Current FUS Phase 0 and Phase I clinical trials primarily focus on the safety and efficacy of ultrasound, both in conjunction with standard-of-care therapies and other experimental treatments (Table 2). In each trial, ultrasound treatment is given directly before or following maximal surgical resection with or without adjuvant chemoradiation with alkylating agent TMZ, as well as other therapeutics, including etoposide (inhibits DNA synthesis), panobinostat (causes cell cycle arrest and apoptosis), doxorubicin (anthracycline), carboplatin (alkylating agent), and pembrolizumab (immune checkpoint inhibitor), among others [116].

### 4.3. Clinical versus Preclinical Advancements

FUS has proven to be a promising technology for enhancing the treatment of gliomas in animal models; however, challenges remain in clinical translation. There is a disconnect between preclinical and clinical glioma research: preclinical models typically focus on treating the primary tumor, whereas the clinical need lies more in post-resection therapies to limit recurrence and enhance adjuvant treatment. Additionally, given the significant degree of cellular and molecular heterogeneity of gliomas, it is challenging to accurately mimic the tumor microenvironment in cell or animal models [119]. Another challenge in HGG treatment and research is that the success of an experimental therapy in preclinical studies is often not predictive of success in human patients. The primary experimental therapies currently being explored for GBM include immunotherapies, such as checkpoint inhibitors and CAR-T cell therapy, viral therapy, angiogenesis inhibitors, and gene therapies. While many experimental therapies have shown promise in preclinical models, few have achieved strong safety or efficacy results in human patients [120].

## 5. Conclusions and Future Directions

Technological innovations and new FUS systems have continued to advance the applications and implementation of FUS-based therapies for infiltrating gliomas. The existence of a multitude of FUS systems and protocols introduces new challenges in the context of the standardization of treatment settings and efficacy metrics. In the initial clinical MB-FUS trials, the primary focus has been the safety and feasibility of treating larger volumes and repeated cycles, as well as early assessments of drug delivery. As FUS technologies evolve toward clinical implementation, it will be essential to develop methods for treatment standardization and correlate treatments with the desired therapeutic effects. Despite the tremendous progress in the field, challenges continue to exist in the translatability and comparability of preclinical studies. The development of preclinical tools and models reflective of clinical conditions will also be highly valuable in the predictive testing of novel therapeutic combinations.

## Figures and Tables

**Table 1:** Clinical FUS systems.

Name	Company	Method of Operation
Exablate model 4000 Type 2	InSightec (Tirat Carmel, Israel)	Multi-helmet—MRgFUS
NaviFUS^®^	NaviFUS (Taipei, Taiwan)	Multi-element FUS
NeuroAccess	Cordance Medical (Mountain View, CA, USA)	Multi-element FUS
Sonocloud9	CarThera (Paris, France)	Implanted device

**Table 2:** Registered clinical trials using FUS for glioma are separated by glioma type. An overview of ongoing clinical trials for treating HGGs.

Study Name	NCT Number	Conditions	Interventions
The Use of Focused Ultrasound and DCE K-trans Imaging to Evaluate Permeability of the Blood-Brain Barrier	NCT04063514	Low-grade glioma	DWL doppler sonography Brainsonix Pulsed LIFU
Study of Sonodynamic Therapy in Participants With Recurrent High-Grade Glioma	NCT04559685	High-grade glioma	aminolevulinic acid (ALA) InSightec ExAblate system
Safety Study of the Repeated Opening of the Blood-brain Barrier With the SonoCloud^®^ Device to Treat Malignant Brain Tumors in Pediatric Patients (SONOKID)	NCT05293197	Glioma	CarThera SonoCloud9
ExAblate (Magnetic Resonance-guided Focused Ultrasound Surgery) Treatment of Brain Tumors	NCT01473485	Glioma	InSightec ExAblate system
Assessment of Safety and Feasibility of ExAblate Blood-Brain Barrier (BBB) Disruption	NCT03551249	Glioma GBM	InSightec ExAblate system
Assessment of Safety and Feasibility of ExAblate Blood-Brain Barrier (BBB) Disruption in GBM Patients	NCT04998864	GBM	InSightec ExAblate system
Blood-Brain Barrier Disruption (BBBD) for Liquid Biopsy in Subjects With Glioblastoma Brain Tumor	NCT05383872	GBM	InSightec ExAblate system
Assessment of Safety and Feasibility of ExAblate Blood-Brain Barrier (BBB) Disruption for Treatment of Glioma [117]	NCT03616860	GBM	InSightec ExAblate system
Safety of BBB Disruption Using NaviFUS System in Recurrent Glioblastoma Multiforme (GBM) Patients	NCT03626896	GBM	NaviFUS System
Sonodynamic Therapy With ExAblate System in Glioblastoma Patients (Sonic ALA)	NCT04845919	GBM	5-ALA InSightec ExAblate system
ExAblate Blood-Brain Barrier Disruption for Glioblastoma in Patients Undergoing Standard Chemotherapy [76,77]	NCT03712293	GBM	Temozolomide InSightec ExAblate system
Safety and Efficacy of Transient Opening of the Blood-brain Barrier (BBB) With the SonoCloud-9 (SC9-GBM-01) [78]	NCT03744026	GBM	Carboplatin CarThera SonoCloud9
Phase 2a Immune Modulation With Ultrasound for Newly Diagnosed Glioblastoma	NCT05864534	GBM Giosarcoma	Balstilimab Botensilimab Liposomal Doxorubicin CarThera Sonocloud-9
Exablate Blood-Brain Barrier Disruption With Carboplatin for the Treatment of rGBM	NCT04440358 NCT04417088	Recurrent GBM	Carboplatin InSightec ExAblate system
Sonodynamic Therapy in Patients With Recurrent GBM (GBM 001)	NCT06039709	Recurrent GBM	5-ALA Neuro-navigation guided LIFU
Efficacy and Safety of NaviFUS System add-on Bevacizumab (BEV) in Recurrent GBM Patients	NCT04446416	Recurrent GBM	Bevacizumab NaviFUS system
Evaluate the Safety and Preliminary Efficacy of the Combination of NaviFUS System With Re-irradiation for rGBM Patients	NCT04988750	Recurrent GBM	NaviFUS sysem
Sonocloud-9 in Association With Carboplatin Versus Standard-of-Care Chemotherapies (CCNU or TMZ) in Recurrent GBM (SONOBIRD)	NCT05902169	Recurrent GBM	Carboplatin Lomustine Temozolomide CarThera SonoCloud9
Randomized Study of Neo-adjuvant and Adjuvant Pembrolizumab With and Without Targeted Blood Brain Barrier Opening Using Exablate MRI-guided Focused Ultrasound (Exablate MRgFUS) for Recurrent Glioblastoma	NCT05879120	Recurrent GBM	Pembrolizumab InSightec ExAblate system
Ultrasound-based Blood-brain Barrier Opening and Albumin-bound Paclitaxel and Carboplatin for Recurrent Glioblastoma [78,118]	NCT04528680	Recurrent GBM	albumin-bound paclitaxel carboplatin CarThera Sonocloud-9
FUS Etoposide for DMG—A Feasibility Study	NCT05762419	Diffuse Midline Glioma (DMG)	Etoposide Neuro-navigator controlled FUS
Noninvasive Focused Ultrasound (FUS) With Oral Panobinostat in Children With Progressive Diffuse Midline Glioma (DMG)	NCT04804709	DMG	Panobinostat Neuro-navigator controlled FUS
Blood Brain Barrier (BBB) Disruption Using Exablate Focused Ultrasound With Doxorubicin for Treatment of Pediatric DIPG	NCT05630209 NCT05615623	Diffuse Intrinsic Pontine Glioma (DIPG)	Doxorubicin InSightec ExAblate system
A Phase 2 Study of Sonodynamic Therapy Using SONALA-001 and Exablate 4000 Type 2.0 in Patients with DIPG	NCT05123534	DIPG	ALA InSightec ExAblate system
